# Aortic enlargement in coarctation is not common in patients without a bicuspid valve: rethinking etiologies

**DOI:** 10.1186/1532-429X-16-S1-P131

**Published:** 2014-01-16

**Authors:** Joel D McLarry, Craig S Broberg

**Affiliations:** 1 Oregon Health and Science University, Portland, Oregon, USA

## Background

Aortic coarctation is considered an endothelial disorder. It is associated with bicuspid aortic valve (BAV), cerebral aneurysms, and aortic dilatation and dissection. Accordingly, guideline statements recommend complete aortic imaging in all coarctation patients. Yet is enlargement mostly seen in those with a coexisting bicuspid aortic valve?

## Methods

Consecutive patients referred for cardiac MRI for repaired aortic coarctation, bicuspid aortic valve, or both were retrospectively reviewed. Patients with prior aortic valve or ascending aortic surgery were excluded, though prior coarctation intervention was not excluded. Maximum ascending aortic dimension was obtained, and averaged for patients with BAV alone, BAV and coarctation, or coarctation alone. Percentage of patients with aortic enlargement defined as a maximum diameter >40 mm were calculated for each group, and appropriate comparisons made between groups.

## Results

A total of 77 patients (58% male, age 36 ± 13.7) were included in the study including 29 with BAV alone, 33 patients with BAV and coarctation, and 15 patients with coarctation alone. Average maximal aortic dimension was 43 mm, 34 mm, and 29 mm in each group respectively (p < 0.001). Additionally, mean aortic diameter was less when comparing the coarct alone and BAV and coarct groups (p = 0.03). Aortic enlargement (>40 mm) was present in 69% of BAV patients, but only 20% of BAV and coarctation patients, and no patients with coarctation alone (p < 0.001).

## Conclusions

In this cohort of patients referred for CMR, ascending aortic enlargement after repaired coarctation is uncommon in the absence of BAV, implying that aortic enlargement is not simply due to adverse pressure loading. The finding argues for a different endovascular physiology in BAV patients compared to those without.

## Funding

None.

**Table 1:** Aortic Coarctation, Bicuspid Aortic Valve, and Aortic Enlargement

	N	Age	N with Aortic Enlargement	% with Aortic Enlargement	Mean Maximal Ascending Aortic Diameter (95% confidence intervals)
BAV alone	29	46 ± 15.3	20	69	43.1 (40.2-46.0)

BAV + Coarct	35	31 ± 8.3	7	20	34.0 (31.5-36.5)

Coarct alone	13	46 ± 15.7	0	0	29.2 (27.2-31.2)

